# THE LONG-TERM LONELINESS OF WIDOWHOOD: A SYSTEMATIC REVIEW OF MARITAL STATUS DIFFERENCES

**DOI:** 10.1093/geroni/igac059.1370

**Published:** 2022-12-20

**Authors:** Anneke Vedder, Jeffrey Stokes, Kathrin Boerner, Henk Schut, Paul Boelen, Bibi Schut, Margaret Stroebe

**Affiliations:** Faculty of Social & Behavioural Sciences, Utrecht, Utrecht, Netherlands; University of Massachusetts Boston, Boston, Massachusetts, United States; University of Massachusetts Boston, Boston, Massachusetts, United States; Utrecht University, Departement of Psychology, clinical psychology, Utrecht, Utrecht, Netherlands; Utrecht University, Departement of Psychology, clinical psychology, Utrecht, Utrecht, Netherlands; Utrecht University, Departement of Psychology, clinical psychology, Utrecht, Utrecht, Netherlands; University of Groningen, Experimental Psychopathology, Department Clinical Psych. & Exp. Psychopathology, Groningen, Groningen, Netherlands

## Abstract

Loneliness can be prominent in bereavement, possibly leading to compromised mental and physical health. We systematically reviewed the extent of loneliness across marital status groups, examining the prevalence, intensity, risk factors, and correlates of loneliness in widowhood, compared to other marital statuses. Studies that met predefined criteria as well as investigated marital status (comparisons) were included in the review. For reporting, we followed the PRISMA statement. Thirty-eight studies were included. Widowhood was associated with a greater likelihood and intensity of loneliness when compared to other marital statuses, and especially the divorced. Widowers were on average lonelier than widows. Findings suggest that , widowed persons are uniquely vulnerable to loneliness, and that, in the long-term, loneliness may be more pronounced among the widowed than the divorced. However, methodological shortcomings (e.g., heterogenous samples, different measures of loneliness) of available studies must be considered, and future research should aim to overcome these limitations.

